# Learning, Memory, and Transcranial Direct Current Stimulation

**DOI:** 10.3389/fpsyt.2012.00080

**Published:** 2012-09-03

**Authors:** Joaquim P. Brasil-Neto

**Affiliations:** ^1^Laboratory of Neurosciences and Behavior, Biology Institute, University of BrasíliaBrasília, Brazil

**Keywords:** tDCS, memory, learning

## Abstract

Transcranial direct current stimulation (tDCS) has been the subject of many studies concerning its possible cognitive effects. One of the proposed mechanisms of action for neuromodulatory techniques, such as transcranial magnetic stimulation and tDCS is induction of long-term potentiation (LTP) and long-term depression (LTD)-like phenomena. LTP and LTD are also among the most important neurobiological processes involved in memory and learning. This fact has led to an immediate interest in the study of possible effects of tDCS on memory consolidation, retrieval, or learning of various tasks. This review analyses published articles describing beneficial or disruptive effects of tDCS on memory and learning in normal subjects. The most likely mechanisms underlying these effects are discussed.

## Introduction

Repetitive transcranial magnetic stimulation (rTMS) and transcranial direct current stimulation (tDCS) are rapidly emerging as potential neuromodulatory tools; TMS already has approved therapeutic applications in neurology and psychiatry (Ragert et al., 2008; Celnik et al., 2009; Hummel et al., 2009; Brunoni et al., 2010).

The after-effects of both rTMS and tDCS sessions are believed to be related to long-term depression (LTD) and long-term potentiation (LTP)-like phenomena (Lømo, 2003; Esser et al., 2006), as well as to induction of gene expression and other mechanisms (Fritsch et al., 2010). During tDCS protocols, a weak current (1 or 2 mA) is delivered by a battery through a pair of electrodes attached to the scalp. In the case of anodal tDCS, the anode is attached to the scalp area to be stimulated and the cathode to the contralateral supraorbital area on the forehead; the arrangement is reversed for cathodal tDCS. It is well established that anodal tDCS increases cortical excitability whereas cathodal tDCS increases the excitability threshold probed with single transcranial magnetic pulses over the motor cortex (Jacobson et al., 2012). Anodal tDCS has been found to depolarize neuronal membranes, while cathodal tDCS induces hyperpolarization. Both carbamazepine, a sodium channel blocker, and flunarizine, a calcium channel blocker, have been found to preclude these effects. Moreover, long-lasting excitability after-effects in either direction can be blocked by dextrometorphane, an *N*-methyl-d-aspartate (NMDA) receptor antagonist (Nitsche et al., 2003).

Studies with rTMS are far more numerous than those with tDCS, but the latter technique is gradually gaining more attention, especially due to its potential advantages: it is less expensive, more portable, and allows for very effective sham stimulation in experimental protocols (Gandiga et al., 2006).

A drug or procedure capable of improving memory in both normal individuals and patients is sort of a “Holy Grail” in medicine. Since LTD and LTP are also strongly involved in memory and learning, a logical hypothesis would be that both rTMS and tDCS would be capable of either disrupting or improving these processes in normal subjects and patients. This possibility has led to the recent publication of many experimental results of neuromodulation of memory and learning processes in both normal subjects and patients.

In this review we will discuss experimental work dealing with potential neuromodulatory effects of tDCS upon learning and memory in normal subjects. It is important to understand how tDCS may affect the normal brain before attempting to use it therapeutically. A clear advantage of tDCS over rTMS in this setting is that it provides a truly sham stimulation to be compared to actual cortical stimulation (with TMS, even especially designed “sham coils” do not evoke the same scalp sensations as real stimulation). tDCS does not evoke any scalp sensations apart from an initial itching while current is being adjusted; this may be replicated by a few seconds of electrical stimulation followed by current interruption during sham sessions. This effectively rules out placebo effects, arousal, enhancement of attention and other non-specific actions of the tDCS procedure (Sparing and Mottaghy, 2008).

## Effects on Learning

One of the earliest and most interesting studies of the effects of tDCS upon memory consolidation and retrieval took advantage of the ease of application and unobtrusiveness of the technique and applied anodal tDCS bilaterally over frontocortical sites every 30 min during sleep periods rich in slow-wave activity, resulting in subsequent improvement in tests of declarative memory (Marshall et al., 2004).

The effect of prefrontal cortex tDCS on implicit learning was also tested in the setting of a probabilistic classification learning (PCL) protocol (Kincses et al., 2004). Ten minutes of anodal tDCS applied to the left prefrontal cortex of 22 healthy subjects while they performed a PCL task improved implicit learning; in contrast, no effect was observed with either cathodal left prefrontal stimulation or primary visual cortex tDCS.

In order to investigate the role of the primary motor cortex (M1) in motor learning, especially in the formation of motor memories, Galea and Celnik (2009) applied anodal tDCS to M1 of nine healthy subjects during motor training. Anodal tDCS was found to increase the magnitude and duration of motor memories.

In another learning paradigm, namely implicit learning of an artificial language, de Vries et al. (2010) found that after 20 min of tDCS applied to Broca’s area during the acquisition of an artificial grammar, subjects performed better in a violation detection task than controls who had undergone sham stimulation or real tDCS to another brain area unrelated to speech.

Another cortical area, the posterior parietal cortex (PPC), has been studied during anodal tDCS in several visual orienting tasks (Bolognini et al., 2010). It has been found that right PPC anodal tDCS, but not left PPC anodal tDCS, enhances visual search skills when applied either by itself or in addition to training.

## Effects on Working Memory

During a verbal n-back working memory (WM) task, as n (i.e., WM load) increases, subjects show poorer behavioral performance. A brief period of practice or even increased familiarity with the task can improve WM performance and lead to activation changes in the PPC in neuroimaging studies. Parietal tDCS was shown to be capable of hampering the improvement in performance, giving further support to the role of the PPC in this kind of task (Sandrini et al., 2012).

On the other hand, Fregni et al. (2005) reported that, on a three-back WM task, 15 normal subjects had significant accuracy improvement in the task during anodal tDCS of the left prefrontal cortex; this could not be explained by slowed responses, since response times were not changed by stimulation. Moreover, cathodal tDCS of the same area or anodal stimulation of the primary motor cortex (M1) had no effect. The authors concluded that left prefrontal anodal stimulation leads to enhancement of WM performance. Their results were later confirmed by other investigators (Ohn et al., 2008; Andrews et al., 2011).

The neurophysiological basis for modulation of WM by left dorsolateral prefrontal cortex was investigated with recording of underlying electroencephalographic activity (Zaehle et al., 2011).

After anodal tDCS, oscillatory power in the theta and alpha bands was amplified and WM performance enhanced; on the other hand, cathodal tDCS decreased alpha and theta oscillatory activity and disrupted WM.

The effect of transcranial random noise stimulation (tRN) of the left dorsolateral prefrontal cortex on a WM task was compared to the effects of tDCS applied to the same region (Mulquiney et al., 2011). While tDCS increased the speed of performance of the two-back WM task, tRN had no effect.

The first study to verify whether left anodal tDCS applied to the DLPC (dorsolateral prefrontal cortex, corresponding to the F3 position of the 10–20 international system for EEG electrode placement) during the persistent performance of a WM task would improve performance on a subsequent WM task to a greater extent that either previous tDCS at rest or cognitive activity by itself was performed by Andrews et al. (2011). The result was that the combination of anodal tDCS applied to the DLPC with a WM task was superior to either tDCS or the cognitive task alone in improving the performance of a subsequent digit span forward task.

Although left prefrontal anodal stimulation increased accuracy without changing response times, bifrontal tDCS has been found to slow reaction times in a WM task (Marshall et al., 2005). More specifically, anodal and cathodal tDCS were applied bilaterally over prefrontal regions, over 15 min repeatedly (15-s-on/15-s-off), while subjects performed a modified Sternberg task. There were also sham tDCS sessions. Under such experimental conditions, reaction times increased linearly with set size, and the slope of such increase was comparable for active and sham stimulation; this was regarded as evidence that the time required for memory scanning had not been affected by tDCS. However, reaction times were slowed during active stimulation as compared to sham tDCS, indicating that real stimulation had impaired neuronal processing related to response selection and preparation.

In contrast to these findings, another series of experiments (Ferrucci et al., 2008) reported no increase in accuracy by bilateral prefrontal tDCS and faster reaction times after cathodal bilateral tDCS of the prefrontal cortices during a modified Sternberg task. The authors explain the discrepancy between their results and those previously described in the literature on the basis of differences in tDCS methodology. In fact, Ferrucci et al. used a non-cephalic reference electrode; there were also differences in wash-out periods and in intensity of stimulation. In addition, however, Ferrucci et al. also tested the effect of cerebellar tDCS during performance of the modified Sternberg test, finding that such stimulation impaired the usual practice-dependent proficiency increase.

The finding that patients with parietal lobe damage may exhibit selective WM impairment in recognition but not in recall tasks was the basis for a study in which normal subjects underwent cathodal (i.e., inhibitory) tDCS to the right inferior PPC and then performed separate blocks of an object WM task probed by recall or recognition. WM was selectively impaired in recognition tasks, as is usually the case for patients with parietal lesions (Berryhill et al., 2010).

## Effects on Memory Retrieval

The role of the temporal lobes on the generation of false memories was investigated by Boggio et al. (2009). Thirty normal subjects underwent one of three stimulating conditions during the acquisition and retrieval phases: anodal left/cathodal right anterior temporal lobe tDCS, left anodal anterior temporal lobe tDCS and sham tDCS stimulation. Both forms of active stimulation resulted in a decrease of 73% in the formation of false memories, without any effect on the veridical ones.

The reports of enhanced visual memory in autism, together with left hemisphere deficit and right hemisphere compensation, led to a study in which normal subjects who had left cathodal (i.e., inhibitory) anterior frontal tDCS in conjunction with right anodal (i.e., excitatory) anterior frontal tDCS showed an improvement in visual memory similar to that described for autistics (Chi et al., 2010).

Fronto-temporal tDCS has been used to probe the specific role of each cerebral hemisphere on the enhancement of memories with different emotional valences (Penolazzi et al., 2010). Right anodal/left cathodal tDCS was found to specifically enhance the recall of pleasant images with respect to both unpleasant or neutral images; conversely, left anodal/right cathodal tDCS favored the recall of unpleasant images over pleasant or neutral ones. This result was interpreted as supportive of the specific-valence hypothesis, which holds that the right cerebral hemisphere specializes in processing of unpleasant memories, while the left hemisphere specializes in the processing of pleasant memories (Penolazzi et al., 2010). Such result, however, is somewhat puzzling, since in most tDCS paradigms anodal stimulation, being excitatory, has been found to improve function (Jacobson et al., 2012), but in this case one would have to interpret its effect as detrimental to the stimulated cortical area in order not to contradict the specific-valence hypothesis. The authors hypothesize, therefore, that excessive stimulation of the underlying fronto-temporal cortex could result in impairment of processing of unpleasant memories in the right hemisphere or of the pleasant ones in the left hemisphere (Penolazzi et al., 2010).

## Discussion

Most tDCS studies performed so far show a consistent positive effect of left DLPC anodal stimulation on WM, when the cathode is applied to the right supraorbital region. This effect may not occur, or even be reversed, with different electrode montages, such as bilateral prefrontal stimulation (Marshall et al., 2005) or use of a non-cephalic cathode (Ferrucci et al., 2008).

Anodal stimulation of cortical areas specifically engaged in learning the task at hand also seem to enhance performance, as in the case of Broca’s area during language tasks (de Vries et al., 2010), the PPC in visual orientation tasks (Bolognini et al., 2010), the primary motor cortex during motor learning tasks (Galea and Celnik, 2009), the prefrontal cortex during implicit learning (Kincses et al., 2004), or even the left DLPC during a persistent WM task (Andrews et al., 2011).

Inhibitory (i.e., cathodal) tDCS has been investigated regarding its ability to disrupt normal cortical physiology. Particularly noteworthy are studies attempting to induce “reversible lesions.” This was the case for the study of cathodal tDCS of the PPC, which resulted in selective WM impairment for recognition tasks (Berryhill et al., 2010) and of both temporal lobes, which reduced the formation of false memories (Boggio et al., 2009). A “savant-like” phenomenon was also induced by frontal inhibitory (i.e., cathodic) stimulation, which resulted in improved visual memory (Chi et al., 2010). However, as recently pointed out in a meta-analytical review of the effects of tDCS polarity in the motor and cognitive domains (Jacobson et al., 2012), it is often difficult to generate cathodal inhibitory effects in cognitive studies.

A noteworthy study which is also not in line with the overall impression that anodic stimulation usually improves function of the underlying cortical area is the one dealing with emotional enhancement of memories (Penolazzi et al., 2010). In order not to contradict the specific-valence hypothesis, one would have to assume, as did the authors of that manuscript, that in their specific paradigm anodal stimulation of the left hemisphere impaired processing of pleasant memories instead of facilitating it. Further studies should therefore be undertaken in order to accept or refuse such assumption.

## Conclusion

Although there are still few studies of the effects of tDCS upon normal physiologic processes underlying learning and memory, most results are remarkably consistent with the experimental hypothesis of a major role for the left DLPC in WM, and also show that WM performance can be enhanced by anodal stimulation over this area. By the same token, anodic stimulation of Broca’s area, the primary motor cortex and other areas primarily involved in specific learning paradigms can also enhance performance. Moreover, there seems to be a summation of the effects of tDCS and training (Andrews et al., 2011); this might well be of therapeutic value in the near future. tDCS has also been proven capable of inducing “reversible lesions” of specific cortical areas, which are useful for disclosing the normal interplay of excitation and inhibition between different cortical areas during learning and memory processes.

Further studies are required to shed light into the intrinsic mechanisms of such effects (Gladwin et al., 2012). Cathodal inhibitory effects, in particular, have not been obtained in many cognitive studies and the reasons for this are still poorly understood (Jacobson et al., 2012). The association of tDCS with neuroimaging studies would be interesting to ascertain that areas under anodic influence are actually stimulated and that those under the cathode are inhibited in different experimental paradigms; TMS could also be used to probe changes in intracortical inhibition and facilitation in response to tDCS. The development of uniform stimulation protocols is also important to allow for direct comparison between studies (Teo et al., 2011).

In the future, after further understanding of its mechanisms of action is obtained and optimal stimulation protocols are developed, tDCS may become a valuable strategy to improve learning in both normal subjects and patients.

Finally, we believe that experiments should be devised to establish how stable these effects are, since beneficial effects on learning and memory will be much more meaningful if they are found to be durable or even permanent.

## Conflict of Interest Statement

The author declares that the research was conducted in the absence of any commercial or financial relationships that could be construed as a potential conflict of interest.
